# Environmental and Physiological Regulation of Reproduction in the Goldfish: Gonadal Development, Maturation, and Spawning Behavior: A Review

**DOI:** 10.3390/ani16050775

**Published:** 2026-03-02

**Authors:** Makito Kobayashi, Eri Iwata, Peter W. Sorensen

**Affiliations:** 1 Field Studies Institute for Environmental Education, Tokyo Gakugei University, Koganei 184-8501, Tokyo, Japan; 2 Department of Natural Sciences, International Christian University, Mitaka 181-8585, Tokyo, Japan; 3 Faculty of Veterinary Medicine, Okayama University of Science, Ikoino-oka, Imabari 794-8555, Ehime, Japan; eri-iwata@ous.ac.jp; 4 Department of Fisheries, Wildlife and Conservation Biology, University of Minnesota, 1980 Folwell Ave., St. Paul, MN 55108, USA; soren003@umn.edu

**Keywords:** goldfish, GnRH gonadotropins, sex steroids, prostaglandin, sexual behavior, spawning behavior, sex pheromones, sexual plasticity, invasive species

## Abstract

The goldfish, a member of the Cyprinidae (minnow family) is an important and relevant model for understanding how fish function and reproduce and can be better managed. Here, we review recent studies of this species and its strengths as a model system. First, we review current understanding of how its neuroendocrine system regulates gonadotropin release, and then steroidogenesis and finally gonadal maturation in both males and females. Endocrine determinants of gender and sexual plasticity are described. Effects of the fish androgen, 11-ketotestosterone, on sexual and spawning behavior of males and females are reviewed. We also describe how several hormones (androstenedione, 17,20β-dihydroxy-4-pregen-3-one, prostaglandin F2α) and their metabolites function as “hormonal pheromones” to synchronize male–female spawning behavior and physiology. Insight provided by the goldfish has enhanced understanding of the neurobiology, toxicology, and comparative physiology of other fishes and provided lessons applicable to the culture of commercial carp and the control of invasive species, as well as conservation.

## Simple Summary

This paper critically reviews the physiological and ecological processes that regulate reproduction in the goldfish, one of the best understood models amongst the fishes. First, we describe how hormonal changes driven by ecological variables are known to be responsible for gonadal maturation and reproductive behavior—and how this applies to other species. Next, we describe how and why goldfish release hormones to the water to function as potent pheromones which mediating many aspects of male–female behavior. Finally, endocrine determinants of sexuality are reviewed. We show how the relatively large body size of this species, its close relationship to many other important species, and the ease with which it can be maintained make it ideal for studies on comparative endocrinology, toxicology, chemical ecology, fisheries management and conservation.

## 1. Introduction

Environmental and physiological regulation of reproductive processes in the goldfish, *Carassius auratus*, have been intensively studied by many researchers for the past half century, making it one of the best understood amongst the fishes. Spawning behavior is especially well characterized [1,2,3,4]. One of the reasons why goldfish reproductive biology is so well understood is that its reproductive activities, like the medaka, *Oryzias latipes* and zebrafish, *Danio rerio* can be manipulated and observed in the laboratory [5,6]. In particular, the goldfish has proven to be an excellent experimental model fish not only for the study of reproductive biology, but also for investigations of comparative endocrinology [7], physiology [8,9], neuroscience [10,11], behavior [12,13], cognitive science [14,15], evolution [16,17,18], development [19,20,21], genomics [22,23], toxicology [24,25], conservation biology [26], environmental sciences [27,28], and chemical communication [12]. The goldfish is also a member the superorder Ostariophysi, the second largest superorder of fishes, making lessons learned from it applicable to many fishes, including the economically important carps which are both a valuable source of food [29,30,31] and important invasive species [32].

The goldfish is one of about 3000 cyprinid species and is closely related to the crucian carps and other Asian carps. The closest relative of the goldfish (*Carassius auratus auratus* or *Carasius auratus*) is the Chinese crucian carp (*Carassius auratus gibelio* or *Carassius gibelio*) [33]. Although there are some crucian carps in Europe and Japan, these have a different heritage. A red color mutant of the crucian carp was found and cultured in China and later exported to other countries. Because of this it could be argued that the scientific name of goldfish should be changed to *Carassius gibelio*, but *Carassius auratus* is still commonly used.

There are many advantages of using goldfish as an experimental model. Fish of various body sizes are commercially available throughout the year from fish farms or pet shops. Body sizes (3–20 cm) are easy to handle and large enough to collect considerable amounts of blood unlike zebrafish or medaka, from which blood sample collection is difficult. Also, we can obtain sexually mature fish of various body sizes from 20 to 200 g in body weight. If you want to have large sample sizes for blood sampling, you can use many small size sexually mature fish or if you want to trace changes in hormone levels of the same individuals, you can collect blood samples repeatedly from the same fish by using large sexually mature fish.

Various methods of hormone administration have also been established for goldfish including intramuscular injection, intraperitoneal injection, silicone tubing (capsule) implantation [34,35], silicone pellet implantation [36], cholesterol pellet implantation [34], administration via rearing water [37], and by feeding [38]. The price of goldfish and cost of installing rearing facilities is also relatively low compared to mammalian models. Being domesticated, goldfish adapt well to new rearing environments, such as transfer from pet shop to laboratory aquarium and from large stock tanks to small experimental vessels, compared to wild crucian carps, *Carassius auratus langsdorfii*, and *Carassius buergeri* subsp.2 [26,39]. Goldfish eat commercial food soon after their transfer into new rearing vessels, and once adapted to new environments, often undergo sexual maturation, ovulation and perform sexual behavior in the experimental vessels. Since goldfish show a strong appetite once adapted to experimental environments, they can be used for the study of feeding behavior [8,40,41,42].

Goldfish have a high tolerance of surgery including ovariectomy [43], hypophysectomy [44,45], and olfactory tract section [8,46]. Individual identification of many fish in the same aquarium is possible by implanting PIT tags into their intraperitoneal cavities [47]. Classical fin clipping is an effective technique for individual identification. Goldfish can bear such surgical treatments without infection.

Gonadal development and maturation of goldfish can be induced and then maintained by manipulating environmental factors, namely water temperature and photoperiod [47]. Thus, sexually mature fish are available throughout the year. Ovulation can be stimulated by increasing water temperature [37,48,49] or injecting with human chorionic gonadotropin (HCG) [50] after which natural spawning between male and female goldfish occurs. Also, spawning behavior can be induced by injecting prostaglandinF_2α_ (PGF), the lipid hormone that controls female receptivity and pheromone release (see Section 3 and Section 6) into males or females, irrespective of gonadal condition [35]. These methods have enabled experiments of various designs for the study of sexual behavior.

In most studies, goldfish of the common variety, or comet variety, have been used because of their availability and low price. On the other hand, one of the benefits of using goldfish for scientific studies is that there are many varieties of goldfish with various types of body shape, eye shape, fin shape, body color, etc. Use of these varieties enables the study of evolutionary biology [16,17,18] and development [19,20,21]. In this article, studies on environmental and physiological regulation of reproduction of goldfish published over five decades are reviewed.

## 2. Seasonal Reproductive Cycles of Goldfish

**Figure 1:**
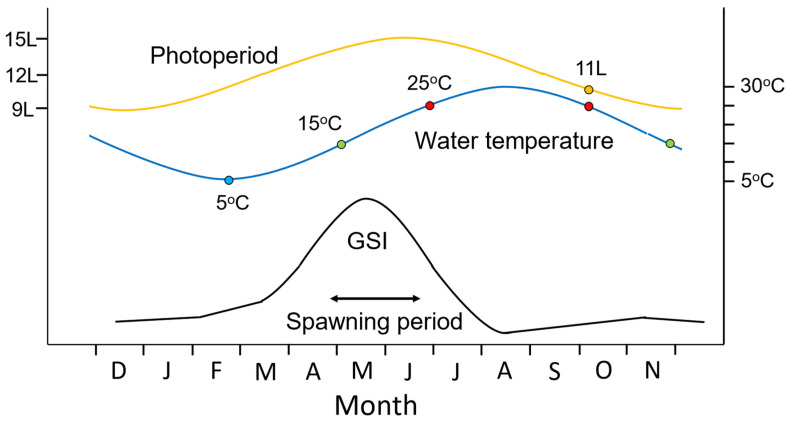
The seasonal reproductive cycle of goldfish in the temperate zone in the Northern Hemisphere. Spawning occurs one or a few times during the spring spawning period when water temperature is rising (blue dotted line) and plants are present. Vitellogenesis starts over 5 °C (blue circle). Ovulation occurs over 15 °C (green circle). Over 25 °C, gonad is regressed (red circles). In the autumn, short photoperiod (11L) suppresses gonadal development although temperature is warm enough (yellow circle). GSI, gonadosomatic index.

**Figure 2:**
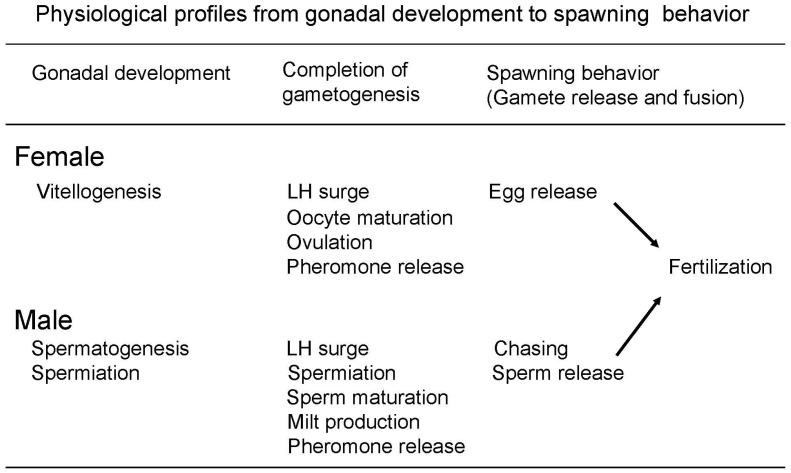
Physiological profiles of female and male goldfish from gonadal development to spawning behavior. LH, luteinizing hormone. See text for details.

The goldfish is a seasonal spawner which has different gonadal stages depending on the season: immature (juvenile fish), maturing (or recrudescent), mature, and regressed [51] which mature at 1–2 years of age (Figure 1). When goldfish are kept under natural temperatures and photoperiod in the temperate zone, they start gonadal development or the early phase of vitellogenesis and spermatogenesis in the winter when the temperature is low. However, continuously low water temperatures in the winter (5–10 °C) will eventually retard further maturation (Figure 1 and Figure 2). In the spring, with an increase in water temperature (10–15 °C), gonadal development shows rapid progress. Although it has been demonstrated in birds that increasing daylength is a major cue to initiate gonadal maturation in spring [52], changes in photoperiod do not affect the gonadal development of goldfish in the spring. When goldfish are kept at 20 °C in spring, gonadal maturation proceeds even under short photoperiod [51]. There is no photoperiodism (i.e., responses to changes in the length of dark phase and light phase) in the spring in the goldfish as has also been demonstrated in other spring spawners, such as bitterling, *Acheilognathus tabira* and honmoroko, *Gnathopogon caerulescence*, and in spring–summer spawners, such as medaka and rose bitterling, *Rhodeus ocellatus ocellatus* [51]. When the temperature is lower than 12 °C, females do not ovulate even though vitellogenesis is completed, probably because the temperature is not suitable for the larval development. When water temperature becomes warmer (15–25 °C), females can ovulate, often several times during the spring spawning period [53]. After the spawning period, the gonad becomes regressed at high water temperatures over 25 °C in the summer.

In the autumn when water temperature decreases (15–25 °C), gonadal development is inhibited by the short photoperiod as also seen in other spring spawners and spring–summer spawners [51]. The inhibitory mechanism by which short photoperiods suppress gonadal development is unknown. In contrast, when goldfish are exposed to a long photoperiod at 20 °C in the autumn, gonadal development proceeds rapidly from a regressed stage to mature stage. This photoperiodism disappears during winter. It has been hypothesized that an inhibition of gonadal maturation in autumn is necessary to avoid producing offspring since larvae would be exposed to cold water with no food in the winter [51]. In the winter, after water temperature goes down (around 5 °C), vitellogenesis starts in the ovary although progress is very slow. It seems that the endocrine system for reproduction does not function in goldfish when temperatures are below 5 °C.

**Figure 3:**
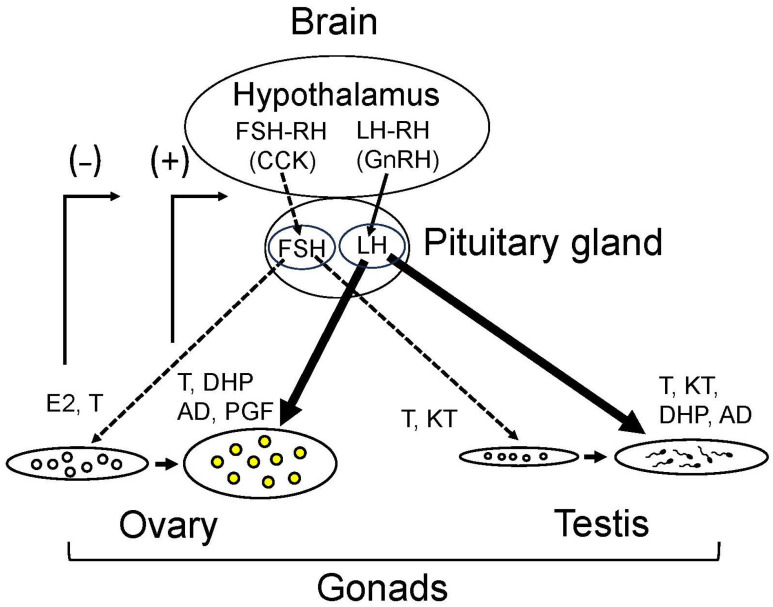
Newly proposed diagrammatic representation of the hypothalamus–pituitary–gonad axis which regulates reproductive activity in teleost fish based on recent studies [56,57,58,59,60]. FSH-RH, follicle-stimulating hormone-releasing hormone; CCK, cholecystokinin; LH-RH, luteinizing hormone-releasing hormone; GnRH, gonadotropin-releasing hormone; FSH, follicle-stimulating hormone; LH, luteinizing hormone; E2, estradiol-17β; T, testosterone; DHP, 17,20β-dihydroxy-4-pregnen-3-one; KT, 11-ketotestosterone; AD, androstenedione. (−), steroid negative feedback on LH release. (+), steroid positive feedback on LH release.

Environmental information, such as water temperature and photoperiod, is received by individual goldfish and converted to endocrine signals by their hypothalamus–pituitary–gonad (HPG) axis (Figure 3). Basal plasma level of pituitary luteinizing hormone (LH) shows a clear correlation with ambient water temperature rather than gonadal maturity. LH level is low in winter and high in summer when the gonad is most regressed under natural conditions [54]. Also, when sexually mature female goldfish are kept at 10, 20 or 30 °C, plasma LH levels show temperature dependency, leading to speculation that FSH regulates enzymatic activities of the gonad [55].

It has been reported that light intensity and changes in day length are received by both the eyes and the pineal gland in fishes. The pineal organ responds to changes in day and night light intensity and short and long photoperiod. The pineal gland produces and releases melatonin during the dark phase. Melatonin is released into the general circulation and plasma melatonin levels show long and short photoperiod patterns in goldfish regardless of water temperature [61]. In contrast, in salmonid fishes, gonadal development starts under a short photoperiod, and it has been hypothesized that the saccus vasculosus and the pituitary gland receive light and are involved in seasonal gonadal development [62,63]. However, it is not clear how the saccus vasculosus and the pituitary gland regulate the HPG axis in these fish.

It is well established that a spontaneous LH surge and ovulation can be triggered by rising water temperatures in sexually mature female goldfish. Interestingly, the ovulatory LH surge and ovulation are synchronized with photoperiod, and a peak of plasma LH and ovulation occurs in the middle of dark phase of the day even if the temperature is raised during light phase or during dark phase [64]. When female goldfish are acclimated to artificially reversed photoperiod, they ovulate in artificial dark phase, not in natural dark phase. These results suggest the occurrence of LH surge and ovulation are timed by biological clock synchronized to photoperiod [48].

## 3. Artificial Control of the Reproductive Activities of Goldfish by Manipulating Environmental Factors and/or Administrating Hormones

The goldfish is a seasonal spawner and environmental conditions for gonadal development and maturation are well established. We can control sexual activity by manipulating these environmental factors. If you need sexually mature goldfish in winter for your experiments, you could obtain sexually mature fish by raising water temperature up to 15–20 °C before natural temperature goes up in spring. Then, fish become mature earlier than with the natural spawning period. Once goldfish reach gonadal maturity, we can maintain maturity by keeping fish at warm temperature (15–20 °C) under long photoperiod. In most cases, when fish are obtained from pet shops where environmental conditions are quite artificial, we cannot estimate whether the fish have photoperiodism or not from their appearance. Then, regardless of possession of photoperiodism, as long as goldfish are kept at warm temperatures (15–20 °C) under a long photoperiod (14–16 L), gonadal development proceeds in a few months and reaches full maturity. In the summer when water temperature is high and the gonad of goldfish is regressed, lowering the water temperature under long photoperiods can advance gonadal development of goldfish, much earlier than natural gonadal development in spring. Maturity can be maintained at warm water temperatures (15–20 °C) and long photoperiod (14–16 L) [53]. Sexually mature goldfish obtained by this method can be used for sexual behavior experiments [47]. When sexually mature females are kept at 12 °C during the winter they may undergo vitellogenesis but do not ovulate. By raising water temperature from 12 °C to 20 °C during the dark phase of the day, the females ovulate spontaneously mostly in the following dark phase [37,48,49].

Ovulation in female goldfish is stimulated by various environmental factors and occurs synchronously with photoperiod. It is known that wild male and female of Japanese crucian carp, *C. buergeri* subsp. 2, which are closely related species to the goldfish, gather in shallow water area in early morning where vegetation is abundant for egg deposition in the spring spawning period. They will often come to spawning grounds on the day following a water temperature increase or water quality change caused by rainfall [65,66]. Water temperature rise and water quality changes seem to be cues for the ovulation in female crucian carp under natural conditions. In the laboratory, sexually mature female goldfish ovulate after water temperatures rise [37,48,49], plants are added [67], or water is replaced in the rearing vessels [53].

The presence of artificial aquatic plants (for egg deposition) is adequate to trigger an LH surge and spontaneous ovulation in female goldfish [67]. Natural or artificial aquatic plants are essential to perform complete spawning behavior between males and females [26]. When female sexual receptivity (and pheromone release) is stimulated by PGF in goldfish (see Section 5) and the plants are removed, only male courtship or chasing are seen as the egg releasing act by females is not facilitated. However, when the plants are returned into the tanks, full spawning behavior resumes immediately.

Oocyte maturation and ovulation can also be induced artificially by injecting gonadotropin-releasing hormone (GnRH) along with dopamine blockers [68], HCG or fish pituitary extracts. In these cases, the occurrence of ovulation does not synchronize to photoperiod, and ovulation occurs with some latency depending on the hormones [50]. Ovulated females previously injected with HCG perform sexual behavior naturally with sexually mature males [69].

Sexually mature males can be obtained by keeping fish at warm temperature under long photoperiods, but if you need a larger amount of milt for your experiments, injection of HCG is quite effective [70] as is addition of the female preovulatory pheromone, 17,20β-dihydroxy-4-pregnen-3-one (DHP) and related steroid metabolites (see below and Section 6 on pheromones). When you need sexually regressed goldfish for an experiment, you can obtain fish with regressed gonad by keeping fish at high water temperature (over 25 °C) or with a reduced ration of food [71].

## 4. Hormones Involved in Gonadal Development and Maturation

As mentioned above, reproductive activity in goldfish is regulated by environmental factors, mostly water temperature, photoperiod in addition to pheromones by the endocrine system via the HPG axis as in other vertebrates (Figure 3). In recent studies of medaka, zebrafish, and Nile tilapia, *Oreochoromis niloticas*, it has been shown that conventional GnRH stimulates LH release and cholecystokinin (CCK) produced in the hypothalamus stimulates follicle-stimulating hormone (FSH) release [56,57,58]. This finding was a breakthrough in fish reproductive endocrinology. Conventional GnRH can be called LH-RH (luteinizing hormone-releasing hormone) and CCK, FSH-RH (follicle-stimulating hormone-releasing hormone). Unlike other vertebrates, teleost fishes have a dual gonadotropin releasing system for the release of LH and FSH. Since goldfish belong to cyprinids as well as zebrafish, it is highly possible that goldfish have this dual GnRH system.

### 4.1. Ovarian Sex Steroids and Prostaglandin F_2α_ in Female Goldfish

**Figure 4:**
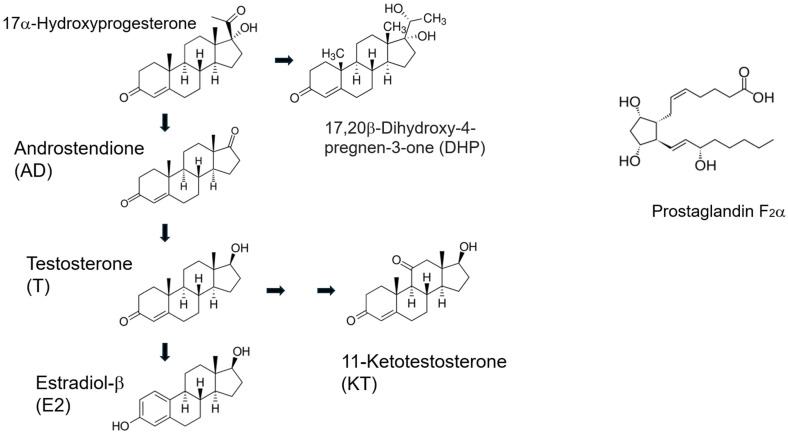
Chemical structures of sex steroids and prostaglandin F2α produced in the gonad of goldfish. Arrows indicate pathways of steroid production.

Being a seasonal spawner, the female goldfish experiences seasonal changes in gonad size and plasma sex steroid levels. Changes in gonadal maturity are typically expressed by gonadal size, GSI [gonadosomatic index (gonad weight/body weight) × 100] and show a peak in spawning period in spring in both females and males (Figure 1) [54,72]. Ovarian follicle cells produce estradiol-17β (E2), testosterone (T), and DHP stimulated by GTH (Figure 4).

**Figure 5:**
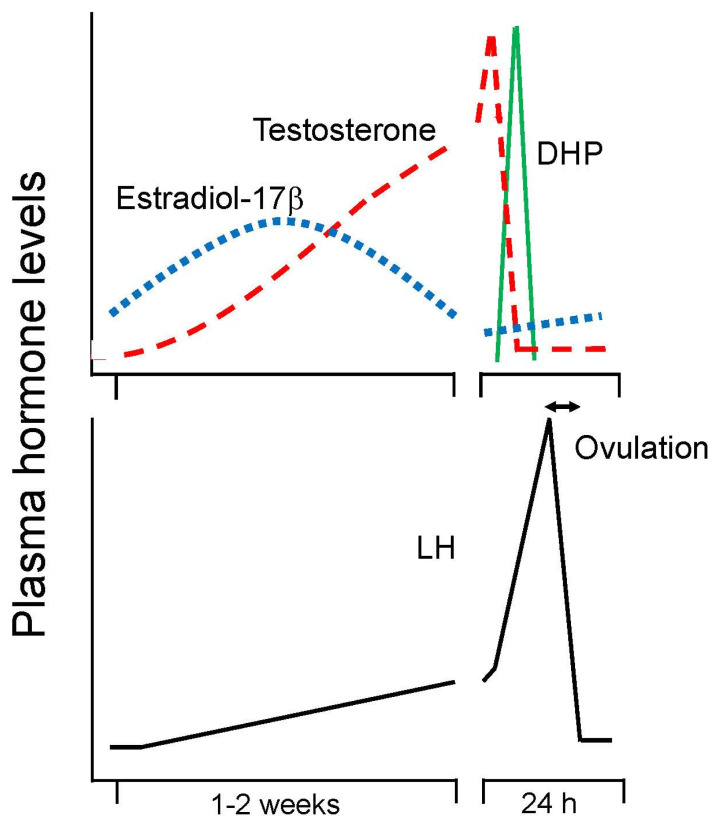
Diagrammatic representation of plasma hormone profiles of the ovulatory cycle in female goldfish during the spawning period. LH, luteinizing hormone; DHP, 17,20β-dihydroxy-4-pregnen-3-one. See text for details.

The teleost ovary produces a large amount of T which is known to be an androgen in mammals. However, androgenic activity of T is negligible in female goldfish. Plasma T level becomes higher than E2 in female goldfish during the spawning period (Figure 5) but females do not show male characters, including secondary characters and male-typical sexual behavior. E2 acts on the liver (the hepatopancreas in the goldfish is a mixed organ of the liver and the pancreas) to induce the production of vitellogenin, the precursor of egg yolk protein.

**Figure 6:**
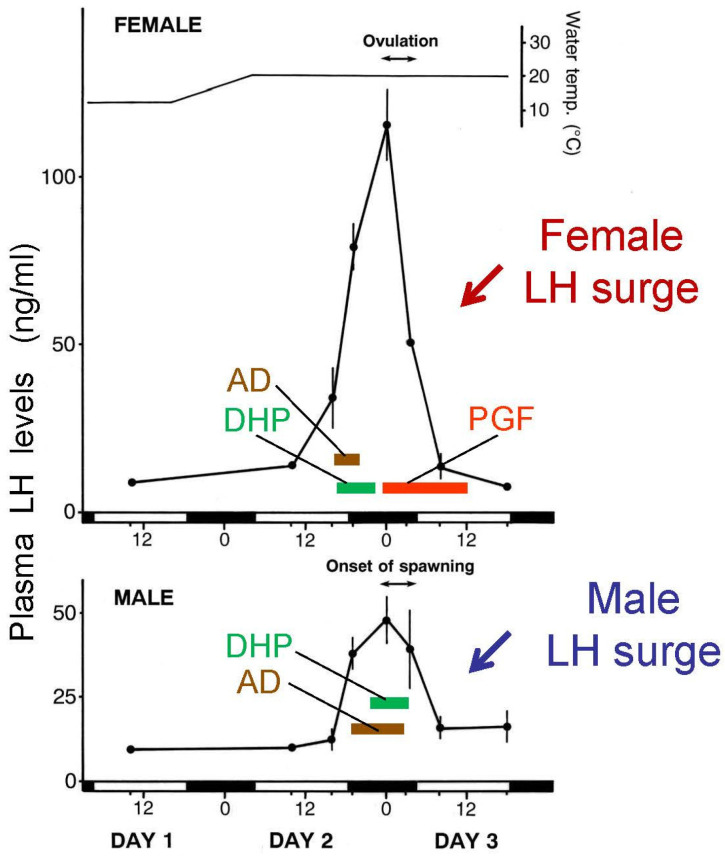
Hormone profiles of female and male goldfish during spawning. Spontaneous ovulation is elicited by a water temperature rise in female goldfish. Rising water temperature stimulates the occurrence of an ovulatory LH surge (release of LH from the pituitary gland) in females which then stimulates the production of DHP and PGF in the ovary. DHP induces oocyte maturation and PGF induces ovulation as hormones. PGF also acts on the brain of the females to elicit the female sexual behavior. DHP and PGF are released into the water as sex pheromones. DHP in the water stimulates male behavior and induces a male LH surge which induces production of DHP and AD in the testis. Circulating DHP in the male stimulates spermiation and milt production for forthcoming spawning. PGF in the water functions as a pheromone to stimulates male sexual behavior. Physiological effects of AD in plasma are unknown in males and females. LH, luteinizing hormone; DHP, 17,20β-dihydroxy-4-pregnen-3-one; AD, androstenedione; PGF, prostaglandin F_2α_.

After vitellogenesis is completed, steroid production in ovarian follicles switches from E2 to T. An increase in plasma T level is considered to be essential for the occurrence of an ovulatory LH surge [73]. At the time of ovulatory LH surge, steroid production switches from T to androstenedione (AD) and DHP, the maturation-inducing steroid (MIS). DHP production is stimulated by the LH surge. Induction of ovulation by an LH surge is a common phenomenon in female vertebrates [37,48] except salmonid species, which show a gradual rise in LH during ovulation and show further increase in LH after ovulation [74]. (Figure 5 and Figure 6). MIS induces oocyte maturation or resumption of meiosis and is released as a pheromone. Later, PGF induces ovulation (or follicular rupture) while inducing female sexual behavior by acting on the brain [45,75].

### 4.2. Testicular Sex Steroids in Male Goldfish

The goldfish testis produces T, 11-ketotestosterone (KT), AD, and DHP in response to GTHs (Figure 4). T and KT are thought to induce spermatogenesis and spermiation [54,72]. Plasma T level is higher than that of KT level. DHP stimulates spermiation and milt production in male goldfish. DHP is transiently produced at the time of spawning, stimulated by the increase in plasma LH level (male LH surge) [49,70].

KT has strong androgenic effects on male goldfish. It stimulates development of male secondary characteristics or tubercles on the opercula and the anterior edge of the pectoral fins. KT is also essential for the occurrence of male sexual behavior. The androgenic effect of T is weak and only when a high dose of T is administered to females, do fish show male-typical characters [76]. KT also induces the increase in red blood cell numbers which serve active spawning behavior of male goldfish (Kobayashi et al., in preparation).

A large amount of AD is produced in the testis (possibly a small amount in the interrenal gland) of goldfish at the time of spawning by stimulation of LH but its physiological role is unknown (Figure 4 and Figure 6). Injection of HCG induces an increase in plasma AD levels (Iwata, unpublished) and release of AD into the water [77]. AD is released in large quantities (100 ng/h) into the water by mature and spawning male goldfish [4,77]. DHP and many other steroids are also released in large quantities by ovulatory females, to serve as pheromones that denote gender and precise reproductive condition (see Section 6 on pheromones). AD pheromone signals the presence of a reproductive male to both males and sexually receptive females [3].

### 4.3. Gonadotropins in the Goldfish

Production of gonadal sex steroids is regulated by pituitary gonadotropins or GTHs (Figure 3). The pituitary gland produces two types of GTH, namely FSH (formerly called GTH-I in fish) and LH (formerly called GTH-II in fish). Until 1990s, fish were only thought to have one LH-like GTH (no FSH), but it is now known that teleost fishes, including the goldfish, have both LH and FSH [78].

GTHs and thyroid-stimulating hormone (TSH) are dimeric glycoprotein hormones with α and β subunits. FSH, LH, and TSH have a common α subunit and hormone specific β subunit. It is known that FSH and LH are produced in the different cells in the pituitary gland in teleost fishes unlike GTHs in mammals where FSH and LH are produced in the same cell [79,80]. In salmonid species, measurement systems for both FSH and LH have been established (RIA or ELISA), and changes in plasma levels of FSH and LH have been measured [81,82]. However, there is only a measurement system for LH in the goldfish and none for FSH so while the former is well understood, the latter is not. (Figure 3, Figure 5 and Figure 6). Problems in establishing a FSH immunoassay system (RIA or ELISA) are due to the difficulty in obtaining enough cyprinid FSH to raise antibodies, and the difficulty of raising antibodies specific enough to detect cyprinid plasma FSH.

### 4.4. Gonadotropin-Releasing Hormone in the Goldfish

#### 4.4.1. Molecular Types of GnRH and Their Location in the Brain

GnRH is a decapeptide produced in the hypothalamus. It was the first identified neuropeptide shown to stimulate the release of FSH and LH in a vertebrate pituitary (Figure 3) [83]. Several molecular types of GnRH peptides have been isolated from the brain of various vertebrates, first in mammals (mammalian type GnRH) and then, two types of GnRH from the chicken (chicken GnRH-I and chicken GnRH-II). GnRH was also isolated from fish and called salmon type GnRH [83]. After the isolation of salmon type GnRH, several other types of GnRH were identified in other fishes by cDNA cloning (Table 1 and Table 2) [84]. These peptides have the generic structure of GnRH: pyrrolic N-terminus, amidated C-terminus, and two regions of conserved amino acid sequence [84]. Some fish species have two types of GnRH and other species have three types of GnRH in their brain (Table 1 and Table 2). Based on their gene locus and molecular phylogeny, GnRHs are presently classified into three molecular types: GnRH1, GnRH2, and GnRH3 [83,84]. These three molecular types have been found in GnRH producing neurons in the hypothalamus (the preoptic area), the midbrain tegmentum and the olfactory bulbs (the terminal nerve ganglion).

GnRH1, also known as gonadotropin-releasing hormone, is produced in the hypothalamus and is known to regulate GTH release. Mammalian GnRH, chicken GnRH-I, and several other types of fish GnRH belong to GnRH1. Most mammalian species have only one type of GnRH, GnRH1 (mammalian type GnRH), but some primates including humans have GnRH1 (mammalian GnRH) and GnRH2 (formerly called chicken GnRH-II) [83]. GnRH2 is produced in the midbrain, and its biological function is unknown. All fish species examined have GnRH2 in the midbrain. GnRH3 (formerly called salmon-type GnRH) is produced in the olfactory bulbs, and these GnRH neurons send fibers to various regions of the brain [85,86]. It has been suggested that GnRH3 functions as a neuromodulator in the various brain areas [87] and not olfactory sensitivity to sex pheromones, at least in the goldfish [88].

The locations of GnRH neurons in the goldfish and salmonid species differ from other fish species which have three types of GnRH (Table 2). Goldfish and salmonid species do not have GnRH1 producing neurons in the hypothalamus, and GnRH2 (chicken GnRH-II) and GnRH3 (salmon-type GnRH) neurons exist in the hypothalamus in goldfish and only GnRH3 in salmonids [83,84]. These GnRH producing neurons send fibers to the pituitary gland. Two types of GnRH seem to regulate LH release in goldfish [86]. In goldfish, GnRH2 and GnRH3 are also found in the olfactory bulbs which send fibers to various areas of the brain and to the eye [85,86].

GnRH2 and GnRH3 peptides in the brain of the goldfish originate mostly from GnRH neurons in the olfactory bulbs. After olfactory tract section, GnRH2 and GnRH3 contents in the brain drastically decrease [85,89] and the immunoreactive fibers in the brain mostly disappear [90]. However, cell bodies of GnRH2 and GnRH3 neurons in the hypothalamus remain as do fibers in the pituitary gland. Olfactory tract-sectioned fish underwent gonadal maturation in males and females and ovulation in females [85,89]. These results suggest that GnRH2 and/or GnRH3 in the hypothalamus are “gonadotropin-releasing hormones”, and GnRH2 and GnRH3 from the olfactory bulbs do not directly stimulate GTH release. The Japanese eel, *Anguilla japonica* and the African catfish, *Clarias gariepinus* have GnRH1 and GnRH2 but do not have GnRH3 and have GnRH1 in the olfactory bulbs (Table 2).

Teleost fish have a unique GnRH releasing system that differs from other vertebrates. Most vertebrates have the median eminence and the portal vessels in the hypothalamus and the nerve terminals of GnRH neurons release GnRH into the capillaries of the median eminence. From there, GnRH is transported into the pituitary cells via the portal vessels. However, teleost fishes lack the median eminence and the portal vessels, and GnRH neurons project the nerve terminals directly into the pituitary gland. GnRHs are released in the pituitary gland. GnRH fibers in the pituitary gland have been observed using immunocytochemistry and GnRHs are detected in the goldfish pituitary gland by radioimmunoassay [85,86]. While it is possible to measure GnRH concentration in the blood of the portal vessels of mammals, it is quite difficult to directly assess the profile of GnRH release in the pituitary gland of teleost fish.

LH release was first confirmed in the goldfish when various types of GnRH peptides and its analogs were administered to goldfish in an in vivo and in vitro pituitary fragment culture system. GnRH peptide showed LH releasing activity [29]. Techniques of radioimmunoassay in combination with HPLC [85,89], immunohistochemistry [86], and in situ hybridization [87] detect the GnRH peptide or GnRH mRNA, but profiles of release of GnRHs in relation to reproduction are not clearly understood.

Another difference between goldfish and mammals is kisspeptin, a 54 amino acid long protein encoded by the KISS1 gene that is known to regulate GnRH in mammals. Goldfish and medaka have kisspeptin in the brain [91], but its role is not clear because knockout of the kisspeptin gene did not affect reproductive activity in the medaka. Furthermore, while kisspeptin producing neurons have sex steroid hormone receptors [92], in vivo administration of kisspeptin peptide has not increased plasma LH in the goldfish [91]. On the other hand, it has been suggested in the chub mackerel, *Scomber japonicus* that kisspeptin stimulates the release of GnRH1 and further gonadal development [93].

Gonadotropin-inhibiting hormone (GnIH) was first identified in the brain of birds [94]. Although studies in GnIH have been conducted in several fish species in relation to GTH secretion and reproduction, the precise function of GnIH remains unclear [95].

In goldfish, dopamine is also known to have an inhibitory effect on LH release. Administration of dopamine antagonist with GnRH, augments LH release [70]. In contrast, serotonin is known to stimulate LH release in goldfish [96].

#### 4.4.2. The Dual GnRH Model in Teleost Fish

Recently, CCK produced in the hypothalamus, a peptide hormone originally identified from the digestive tract, was identified as FSH-RH in the medaka [56], the zebrafish [57], and the tilapia (Figure 3) [58]. It is very possible that the goldfish also has CCK as an FSH-RH because zebrafish and goldfish are closely related species, but this has yet to be determined. Nevertheless, it has been shown that LH producing cells have GnRH receptors and FSH cells have CCK receptors in the pituitary gland of zebrafish. It is interesting that fish have discrete regulatory systems for FSH and LH release, unlike mammals in which FSH and LH are produced in the same cells, and their release is regulated solely by single GnRH [56,57,58,59,60]. It has been shown that administration of GnRH stimulates LH release in the goldfish [29]. In the red seabream, *Pagrus major*, administration of GnRH stimulated LH release but not FSH release [97], suggesting that FSH release is regulated not by GnRH but by CCK.

Notably, the nomenclature for GnRH is rather confusing in teleost fishes. There are three molecular types of GnRH in vertebrates: GnRH1, GnRH2, and GnRH3 and three different areas with GnRH neurons in the brain, the hypothalamus, the midbrain, and the olfactory bulbs. GnRH produced in the hypothalamus is a regulator of GTH release, but the molecular types of actual ‘gonadotropin-releasing hormone’ differ among species. Indeed, according to recent studies, conventional GnRH peptide in the hypothalamus could be called LH-RH and CCK as FSH-RH in teleost fishes [59,60]. In goldfish, GnRH2 and GnRH3 peptides produced in the hypothalamus could be called LH-RH2 and LH-RH3 peptides, respectively. Thereafter, in this paper, we use the terms LH-RH (GnRH) and FSH-RH (CCK) for gonadotropin-releasing peptides which are involved in LH and FSH release.

## 5. Hormonal Regulation of Gonadal Development and Maturation

### 5.1. Females

In female teleost fish, oogenesis starts with mitotic proliferation of oogonia. When oogonia become surrounded by somatic follicular cell layers, the granulosa cell layer and theca cell layer (folliculogenesis) start meiosis and are called oocytes. After hypophysectomy in goldfish, the most advanced stage of oocytes in the ovary is the late perinucleolus stage and oocytes did not undergo vitellogenesis (Figure 3) [44]. These results indicate that GTH is essential for further progress of oocyte development or vitellogenesis. Although meiosis starts in the ovary, it is arrested at the prophase of the first division and vitellogenesis occurs during this stage. Pituitary FSH is thought to stimulate production of E2 in the ovarian follicle cells and E2 acts on the liver to produce vitellogenin. With an increase in vitellogenin incorporation, the oocyte diameter increases [59,60].

Based on the studies on plasma GTH profiles in salmonid fishes, ovarian development (vitellogenesis) is stimulated by FSH and ovarian maturation (development of maturational competence, oocyte maturation, and ovulation) is induced by LH and the MIS [81,82]. Since knock out of FSH-RH (CCK) gene blocked vitellogenesis in female medaka, FSH seems to be essential for vitellogenesis in teleost fishes. After vitellogenesis, the oocytes are not sensitive to the MIS. In the studies of some marine fishes, it has been shown that LH, but not FSH, develops maturational competence of the oocytes [98,99]. Maturational competence can be defined as responsiveness to MIS or development of membrane receptors to the MIS in the oocytes [100,101]. It is thought that goldfish have the same system of development of maturational competence as do the marine fishes. Identification of membrane steroid receptors was a breakthrough in the field of endocrinology since steroid receptors had only been previously identified as nuclear receptors. Interestingly the membrane steroid receptor was first identified from fish oocyte [100]. Now, it is known that goldfish also have membrane receptors for pheromonal sex steroids on their olfactory receptor neurons [102] with different DHP receptors found on the goldfish oocyte [101].

After vitellogenesis in goldfish, production of sex steroids switches from E2 to T due to a decrease in aromatase activity in the follicular cells (Figure 5 and Figure 6) [103]. This increase in plasma level of T is essential for the occurrence of an ovulatory LH surge [73]. Raising water temperature from 12 to 20 °C, triggers an ovulatory LH surge in sexually mature female goldfish. Interestingly, when ovariectomized or sexually regressed females are implanted with T and exposed to a rise in water temperature, these fish show an LH surge synchronized to photoperiod as do sexually mature ovulatory females [73]. These results indicate that T plays a role of positive feedback for the occurrence of the ovulatory LH surge.

As a consequence of a LH surge, ovarian follicles produce DHP and PGF which induce oocyte maturation and ovulation, respectively [104]. Oocyte maturation can be defined as the resumption of meiosis and DHP expedites meiosis up to meta phase of the second meiotic division. Meiosis of oocytes is completed after fertilization or entry of sperm into the oocyte. After ovulation, germ cells are called ova or ovulated eggs in fish. Oogenesis in fish is described in detail by Reading et al. [105].

DHP acts on the membrane receptors of goldfish oocytes as a hormone [100,101] and is subsequently released into the water along with other steroids from the gills as sex pheromone (preovulatory pheromone) which elicits male LH surge in sexually mature males [106] (see Section 6). Pheromonal DHP also stimulates weak chasing of males to females [107]. Internally, DHP actions on the membrane receptor of oocytes accelerate the process of oocyte maturation by stimulating production of the maturation-promoting factor (MPF), cdc2 kinase and cyclin B in goldfish. DHP also activates the formation of preexisting cdc2 kinase and newly synthesized cyclin B. Activation of MPF (production of complex of cdc2 kinase and cyclin B) proceeds oocyte maturation from meiotic metaphase to meiotic anaphase [108].

Ovulatory LH also indirectly stimulates the production of PGF in the follicular cells for ovulation. After ovulation, ovulated eggs in the ovarian cavity stimulate the production of PGF by their presence in the oviduct [75,109]. Circulating PGF also acts on the brain to trigger female-typical sex receptivity and behavior [75]. Finally, PGF and its metabolites are released into the water via urine to function as a sex pheromone (post-ovulatory sex pheromone) which induces male-typical sex behavior or chasing and the sperm releasing act in males [110] (see Section 6).

Similar to many other vertebrates, steroids are known to have both positive and negative feedback on LH release in the goldfish [73,111]. Sex steroids weakly suppress the release of LH during vitellogenesis (negative feedback). After ovariectomy, plasma LH levels rise because of the removal of an inhibitory effect of sex steroids on LH release while replacement therapy of sex steroids lowers the plasma LH levels [111]. After ovariectomy, mRNA levels of FSHβ subunits show a large increase in the pituitary gland but not LHβ subunit levels. The increased levels of FSHβ subunits are suppressed by sex steroids [43].

Negative feedback effects of sex steroids on LH and FSH release have also shown in salmonid species. In the coho salmon, *Oncorhynchus kisutch*, presence of negative feedback of gonadal steroid on LH and FSH release has been shown in both males and females. After gonadectomy, plasma levels LH and FSH increased 5- to 60-fold over pre-surgery levels [112].

### 5.2. Males

The testis of goldfish consists of many seminal lobules with a tubular structure which has a blind end and an opening to the efferent duct. In the lumen of the seminal lobule, many cysts attach to the wall of the lumen. Each spherical cyst is composed of flat and thin shaped Sertoli cells like a soccer ball. Inside the cysts, testicular germ cells develop under the regulation of hormones. “Spermatogenesis” is defined as the entire process by which male germ cells develop from spermatogonia to mobile spermatozoa: spermatogonia, spermatocytes, spermatids, and spermatozoa. Spermatogonia proliferate by mitotic division and spermatocytes start meiotic division. The process by which germ cells develop from spermatids to spermatozoa is called spermiogenesis. Then, spermatozoa are released into the lumen of the seminal lobule by breaking the cystic form of the Sertoli cells. This process is biologically called ‘spermiation’. The term is used in different ways. When expressible milt (spermatozoa and seminal fluid) is observed in sexually mature male fish by applying gentle pressure to the abdomen, it is often said that spermiation has been observed. After spermiation, spermatozoa are not motile, and by increasing the pH of seminal fluid from 7.5 to 8.5, spermatozoa acquire motility [113]. The process of acquisition of motility of sperm is called “sperm maturation”, although there is no morphological change. Spermatozoa which have acquired motility move through the efferent duct and sperm duct (vas deferens). Finally, spermatozoa are released into the water by spawning males. Interestingly, spawning activity itself greatly enhances sperm production as well as enhancing its motility in the goldfish, seemingly via a unique neuroendocrine mechanism associated with an LH surge [114,115]. In goldfish spermatozoa start to swim once in fresh water but not in seminal fluid, saline or sea water. Decrease in ambient osmolarity triggers swimming of the spermatozoa [116]. Spermatogenesis in fish is described in detail by [117].

In studies of salmonids, plasma FSH stimulates the production of androgens, such as T and KT which stimulate early phase of spermatogenesis [81,82]. After a decrease in FSH, plasma LH levels increase and LH stimulates the production of DHP. These hormone profiles suggest that FSH stimulates the early phase of spermatogenesis and LH induces testicular maturation in this group. It is questionable whether FSH is involved in spermatogenesis in goldfish. In studies of medaka, knock out of the FSH-RH (CCK) gene did not suppress the development and maturation of testis, but testicular size was a little smaller than that of control fish. Unlike female medaka in which FSH is essential for vitellogenesis, testicular development is regulated by FSH and LH, and without FSH, LH seems to produce androgens for spermatogenesis in teleost fishes. Interestingly, administration of KT induces all morphological stages of spermatogenesis in both the goldfish [118] and Japanese eel (from spermatogonia to spermatozoa, sperm maturation was not confirmed) [119]. The LH-RH (GnRH), LH and KT system may be a major system for spermatogenesis in goldfish. When male goldfish are mated with ovulatory females, males show a LH surge initially stimulated by female DHP pheromone [106] and later enhanced by the behavioral act itself (Figure 6) [114]. The male LH surge in turn stimulates the production of DHP in the testis. Although KT induced development of whole stages of spermatogenesis, DHP produced by LH surge is thought to boost spermiation, sperm maturation, milt production (testicular hydration) and increase in seminal plasma pH for higher fertilization rate at the time of spawning [4,113]. Injection of HCG induced an increase in plasma DHP [70], and DHP administration induced spermiation in male goldfish [120]. Also, administration of HCG increases milt volume within 24 h in this species [70].

### 5.3. Production of Recombinant Goldfish GTHs Using Silkworm Larvae as a Host

The role of GTHs in gonadal development and maturation in the goldfish has been clearly established by a variety of studies using recombinant GTHs (rGTHs). Because the role of LH is confounded by temperature and there was no direct evidence that FSH is involved in gonadal development. Recombinant goldfish FSH and LH have been produced by using silkworm larvae [71]. Bacteria, such as *Escherichia coli*, do not have the ability of posttranslational modification of proteins, such as glycosylation and subunit dimerization so were not used. Culture systems of amoeba [121], yeast [122], fish egg [123], and CHO cells [124], which are eukaryotes, can be used for the production of glycosylated GTHs but are inefficient. Accordingly, silkworm larvae which can produce a large quantity of glycosylated rGTHs have been used [125]. The authors have prepared two types of rGTHs, dimeric rGTHs and single-chain rGTHs. Since dimeric GTHs lose their biological activity when α and β subunits are dissociated, we designed single-chain rGTHs which α and β subunits are connected and expressed as a single strap of the protein. We expected the single-chain rGTHs would remain in the blood for a longer time without subunit dissociation. Also, production of the single-chain rGTHs is theoretically simpler and easier [71,125]. Interestingly, there seem to be no clear differences in the biological activities between recombinant FSH (rFSH) and recombinant LH (rLH) [71,125]. Both rFSH and rLH stimulate the increase in plasma T levels in male and E2 levels in female goldfish. Both rFSH and rLH also induce milt production in male goldfish. In a small cyprinid species, the rose bitterling, rFSH and rLH induce oocyte maturation and ovulation [71,125]. When goldfish rFSH or rLH have been administered to sexually immature Japanese eel, spermatozoa were observed in the testis of rLH-injected fish and spermatids in rFSH-injected fish. The testis of saline-injected control fish had only spermatogonia [126]. When rFSH and rLH were administered to male and female goldfish, they did not appear to have different effects. Perhaps due to temporal differences in release from the pituitary gland, FSH and LH play different roles in gonadal development and gonadal maturation.

## 6. Hormonal and Pheromonal Regulation of Spawning Behavior in the Goldfish

### 6.1. Spawning Behavior of Goldfish

**Figure 7:**
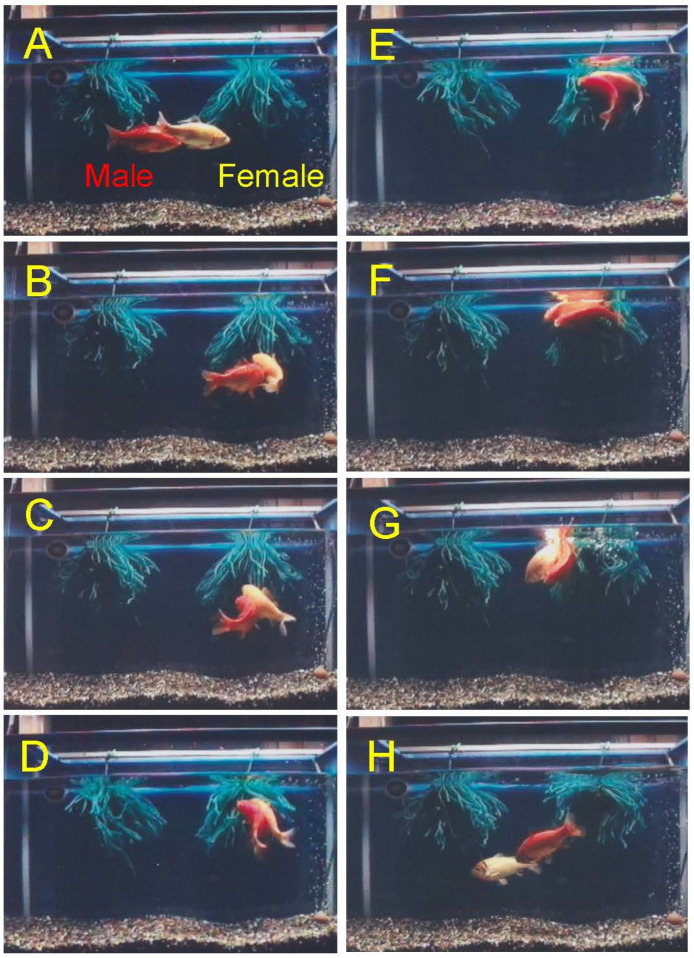
A series of photographs showing spawning behavior of goldfish. (**A**) An ovulatory female goldfish (orange color) releasing sex pheromones which attract a sexually mature male (red color). The male is chasing (courtship) the female. (**B**–**D**) Rise and entry into the floating artificial vegetation made with acrylic yarn. (**E**) Turning on their sides. The male always positions underneath of female (**F**), which releases eggs while the male releases sperm. (**G**) Males and females flip their tails to force eggs release and agitate the water resulting in increase in fertilization rate of eggs and sperm. (**H**) Falling and chasing again. It took 2.2 s from the position shown in photograph (**A**) to the position shown in photograph (**H**).

Physiological regulation of goldfish spawning (sexual) behavior has been intensively studied and is one of the best understood systems amongst the fishes [1,2,3,4]. We summarize our understanding here. Under natural environmental conditions, the female ovulatory LH surge is synchronized with photoperiod, so plasma LH levels show a peak in the middle of dark phase of the day (Figure 6). Consequently, females ovulate and start spawning at midnight under dim light. Male chasing starts in early phase of dark phase (probably triggered by the preovulatory pheromone, see below) and spawning between males and females start just after ovulation in females (Figure 7). Male spawning behavior is characterized by chasing and nudging and culminates in the spawning act (sperm releasing act) [1,2]. Male chasing is persistent and interspersed with the spawning acts (gamete release). Spawning acts are initiated by ovulated (or PGF-injected) females entering floating aquatic plants while the male follows the female. The female and the male(s) then turn on their sides and swim quickly through the plant, releasing eggs or sperm, while flipping their tails, perhaps to help mix the gametes. The male always positions itself underneath—to the side, and in contact with the female during this act. Released eggs are sticky and quickly adhere to the plant. Female spawning behavior continues until all ovulated eggs are released; this may involve hundreds of spawning acts over the course of several hours. Males compete for females by pushing each other during this time [1,2,127].

Ovulation and natural spawning behavior can be stimulated by raising water temperature and adding aquatic plants which stimulates the occurrence of an ovulatory LH surge and then ovulation and PGF production. Using this method, one can observe natural spawning acts and the natural hormonal profile in spawning goldfish [49]. However, it is rather hard work for observers at night. Then, we can induce ovulation of female goldfish by injecting hormones, such as HCG, and change the time of ovulation to observe spawning behavior during the daytime [50]. Alternatively, fish (including male and non-ovulatory sexually immature or sexually regressed female goldfish) can be injected with PGF as this treatment will cause them to perform normal female spawning acts with sexually mature males in several minutes although eggs are not released in this case [8]. Males do not distinguish between ovulated females and PGF-injected females. Thus, one can observe and study spawning behavior throughout the year at any time of the day.

For goldfish spawning, aquatic plants (natural or artificially made with acrylic yarn) are necessary to perform spawning behavior [26]. Without plants, ovulated female cannot perform the egg releasing act, and male chasing continues until females let ovulated eggs leak on the bottom of aquaria. In the case of PGF-injected females without aquatic plants, females are chased until injected PGF is fully metabolized and not effective [26,75].

For behavioral experiments, we usually place one male and one female in experimental vessels to keep experimental designs simple, but crucian carps and goldfish are known to be batch spawners, and several males will normally chase and compete for one ovulated female for spawning [65,66].

### 6.2. Pheromones

#### 6.2.1. Introduction to Pheromones

Pheromones, chemical signals that animals release to the environment to communicate with others of the same species [128], are intimately involved with many aspects of goldfish behavior, physiology, and spawning. Indeed, reproductive hormones and pheromones are one in the same in this species—and almost certainly many others. Four decades of study have now revealed that the goldfish uses several mixtures of hormonal products in combination with other products to function as powerful “hormonal pheromones” which synchronize many aspects of male and female reproductive physiology and behavior. So important are hormonal pheromones to this species that male goldfish which lack a fully functional olfactory system (cranial nerve #1) typically fail to reproduce [8]. Studies of the goldfish hormonal pheromone system have sparked similar studies in dozens of other fishes, revealing that many thousands of species from a variety of taxonomic groups also employ hormonal pheromones (see [4,8]). Here, we briefly review key aspects of the goldfish while focusing on recent work. Several other more detailed reviews of goldfish sex pheromones may be found elsewhere [1,4,129].

It makes good sense that the goldfish and its relatives should have evolved to use hormonal products as pheromones because hormones are produced and cleared/released at highly relevant times and goldfish rely on tight reproductive synchrony to reproduce successfully. Because of these aspects of goldfish biology and the ease with which goldfish behavior and physiology can be studied, the goldfish is arguably the leading model for understanding sex pheromone function in teleost fish, and perhaps all vertebrates. Many Ostariophysans including carps of economic and/or ecological significance have very similar endocrine systems to the goldfish and now appear to use hormonal pheromones in very similar fashions which can be used in culture and/or management [32,130,131]. Here, we briefly review the six pheromones currently known to be employed by goldfish. We describe each briefly, and then elaborate on the four (iii–vi) reproductive pheromones that are most directly associated with spawning:

(i). Alarm Cue. Like all ostariophysans, goldfish release an alarm pheromone when their skin is damaged which alerts conspecifics to danger [132]. It has not been chemically identified in goldfish.
(ii). Species-Identifying Pheromone. All life stages (juvenile, mature) of goldfish as well as both genders of goldfish release an odorous multi-component cue which identifies species [133]. This pheromone (which arguably might be termed a “cue”) appears to comprise multiple polar and nonpolar components which act in concert. The novel bile acid, cyprinol sulfate, and several amino acids appear to have a role in this pheromone but do not comprise the entire signal (Sorensen, in preparation) [3,134,135].
(iii). Male Sex Pheromone. Sexually mature male goldfish release a cue comprising AD and perhaps other androgens which signal the presence of a mature male and evokes inter-male aggression as well as interest from females [77,136]. This signal is described in greater detail below.
(iv). Female Recrudescence Pheromone. Vitellogenic female goldfish release a sex pheromone that attracts mature males. While the identity of this signal is unknown, its release can be stimulated by E2 treatments [1].
(v). Female Pre-ovulatory Sex Pheromone. Ovulatory female goldfish release a changing mixture of sex steroids and their conjugates associated with the LH surge they experience the day oocyte maturation and ovulation. These steroids include the MIS, DHP which serves as a potent priming pheromone that stimulates male, and likely female, endocrine systems as well as male arousal [4,106,127]. This hormonal “priming” pheromone (i.e., a pheromone with largely physiological actions) is explained in detail below.
(vi). Ovulated Female Sex Pheromone. Ovulated female goldfish release PGF along with several of its metabolites which together function as a potent “releasing” pheromone (i.e., a pheromone with primarily behavioral actions) that attracts and stimulate males to spawn [75,110]. This hormonal releasing pheromone is described in detail below.

As described above, the goldfish, like many fish, spawn once or maybe a couple times each year, seemingly as a predator swamping strategy to reduce egg predation by overwhelming predators with large numbers of eggs [4]. This activity takes the form of brief, but highly synchronized events during which time eggs are attached to aquatic vegetation in low light/daybreak (Figure 2). Thus, it is reasonable for crucian carp/goldfish to rely heavily on chemical communication to tightly synchronize male–female physiologies and for spawning behaviors in the darkness. So important are pheromones that anosmic males typically fail to spawn in the laboratory (see Section 7). Goldfish do not appear to use visual cues such color or body shape (Kobayashi, unpublished). When males are aroused and chasing females, they frequently inspect the female’s urogenital aperture and gills, presumably to detect female pheromones which are released by these routes—and thus determine conspecific gender and reproductive condition. They are closely related crucian and common carps, which are also group spawners known to detect/use the same hormonal cues, and their males will also often chase one ovulated female and compete to get best position to spawn with the ovulated female [65,137]. Large groups of fish typically spawn at the same time as the timing of female ovulation is synchronized [4]. It is now clear that the entire process associated with spawning is tightly coordinated by a multi-component hormonally based sex pheromone system that facilitates all components of what is an elegant synchrony between male and female gonadal maturation and behaviors. We briefly describe it below.

#### 6.2.2. Sex Pheromones

**Figure 8:**
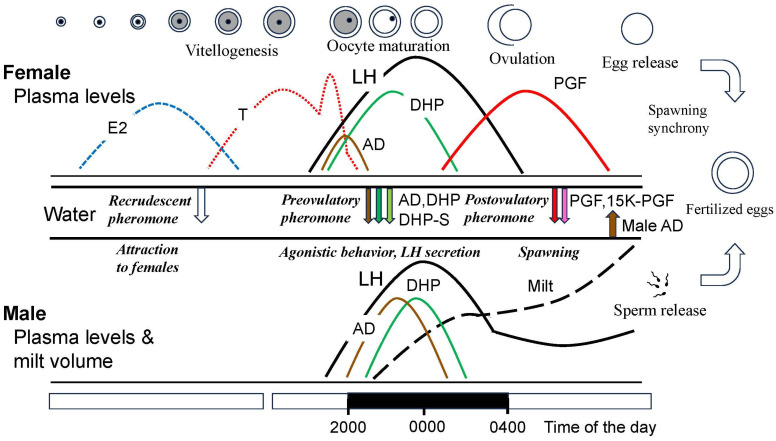
The entire hormonal sex pheromone system in goldfish as presently understood [1,3,4]. The top panel represents the female reproductive system while the bottom is the male. Arrows extending below and above the panels represent pheromones released into the water. Females start to mature and undergo vitellogenesis while E2 rises and causes recrudescent pheromone release. Steroid production switches from E2 to T after vitellogenesis. Later, LH surges. This is associated with an initial increase in androgen production (T and AD) (and release) although this soon decreases as DHP rises to drive oocyte maturation while being released as part of the preovulatory priming pheromone which when detected by males stimulates LH (and AD) release as a pheromone that drives male–male agonistic behaviors. Later DHP decreases and DHP-S is released as a pheromone that stimulates male interest. After ovulation, PGF is produced to stimulate female sexual receptivity and ovulation as a hormone and PGF and 15K-PGF are released to stimulate male behavior as a pheromone. E2, estradiol-17β; T, testosterone; LH, luteinizing hormone; DHP, 17,20β-dihydroxy-4-pregnen-3-one; DPH-S, 17,20β-dihydroxy-4-pregnen-3-one sulfate; AD, androstenedione; PGF, prostaglandin F_2α_.; 15K-PGF, 15keto-prostaglandin F_2α_.

As previously mentioned, when exposed to appropriate stimuli which include a rising temperature, the presence of aquatic plants (oviposition substrate), and very likely pheromones (DHP) (Figure 8), female goldfish experience a preovulatory LH surge which drives steroidogenesis and oocyte maturation so that ovulation occurs within about 12 h. Concurrent with this hormonal surge, female goldfish rapidly clear ovarian steroids to the water. Remarkably, male and female goldfish have developed acute and highly specific olfactory sensitivities to approximately half a dozen of these steroidal products which they then employ as potent and highly relevant sex pheromones. Hormones and pheromones are thus one and the same in goldfish and it is highly adaptive. Although many details of this system still need to be explored, the process seemingly commences as ovulatory goldfish ramp up production and release of DHP via their gills, while levels of circulating androgens including AD fall, as does their release [138]. DHP and AD release can each approach 100 ng/h [138,139]. Both of these free steroids are detected with extreme specificity and sensitivity (10^−13^ and 10^−11^ M thresholds, respectively) by the goldfish olfactory system which has neurons specialized for their detection [140] and employs specialized neural pathways that run to the preoptic area where they synapse with LH-RH (GnRH) and dopamine systems associated with the gonadotropic cells (see [4,88]). Within minutes of detecting DHP, male goldfish experience a substantial LH surge themselves which parallels that of ovulatory females and leads to increased testicular production of DHP, so by morning (spawning) exposed males have over an order of magnitude more sperm (milt) which is also more mobile—a tremendous reproductive advantage [115]. This pheromone also appears to stimulate LH surges in conspecific females, creating a social network so entire groups of goldfish can spawn en masse at the same time to drive predator swamping [4] (Figure 7 and Figure 8).

Although DHP is the primary component of the preovulatory pheromone, it is not the only component. Like many insect pheromones, this pheromone has multiple components which modulate and amplify the effects of DHP. However, in this case, their composition shifts as females undergo oocyte maturation and their steroid profiles change. While it has been argued that fish hormone systems and metabolic pathways may have evolved in ways to accommodate pheromone production [4], this question is unresolved except perhaps for PGF (see below). A second component of the preovulatory pheromone is AD (which is also a component of the male pheromone), which is released by females early in their LH surge and dampens the effects of DHP, perhaps sharpening male–female synchrony [127]. As the LH surge progresses, AD production and release increases but eventually moderates, and another steroid detected by the olfactory system, sulfated 17,20β-dihydroxy-4-pregen-3-one-20 sulfate (DHP-S), becomes of greater importance. This steroid, which is also discerned by its own olfactory receptors and neurons [140], functions as a strong behavioral stimulant and attractant in the hour or so before donor fish ovulate [141]. Its release rate can also exceed 100 ng/h and it is found in the urine, further enhancing its effects and those of DHP [141]. Males with blocked olfactory systems do not experience a LH surge when placed with ovulatory females [69]. The preovulatory pheromone may well have other components and is probably discerned within the context of the species-identifying cue. However, release of all three steroids that comprise the preovulatory pheromone collapses at the time of ovulation when other factors come into play as described below.

**Figure 9:**
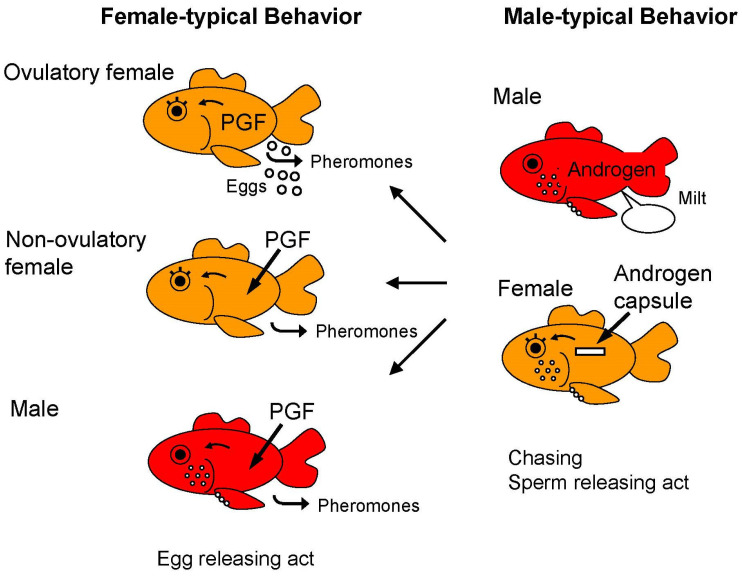
Diagrammatic representation of sex-typical (homo-typical) and hetero-typical sexual behavior in goldfish. An ovulatory female performs female-typical sexual behavior (egg releasing act) by PGF (prostaglandin F_2α_) produced in the ovary. PGF is released into the water as a sex pheromone to stimulate sexually mature males. The male performs male-typical sexual behavior (chasing and sperm releasing act) in the presence of testicular androgen and PGF pheromone from the female. When sexually immature or regressed females (non-ovulatory females) are injected with PGF, the females perform female-typical sexual behavior (egg releasing act) by the effect of PGF. However, actual egg release is not accompanied in this case. Injected PGF is released into the water as a pheromone and attracts sexually mature males. The male performs male-typical sexual behavior with the nonovulatory females. It seems that the male actually releases sperm. When sexually immature or mature males are injected with PGF, the males perform female-typical sexual behavior (egg releasing act) with other sexually mature males. The PGF-injected males do not release eggs, and it is not clear whether the males release sperm instead of eggs by the egg releasing act. When sexually immature, mature or regressed females can be implanted with an androgen capsule in the body cavity. A few days after the implantation of the capsule, the females start to show response to PGF pheromone and perform male-typical sexual behavior (chasing and sperm releasing act) with PGF releasing females. These females do not release sperm or eggs. Sexually mature males and androgen-implanted females develop male secondary sexual characters or tubercles on their opercula and the edge of pectoral fins (small white projections). Eyelashes indicate that the fish is female although this is not biologically true because goldfish do not have eyelashes.

**Figure 10:**
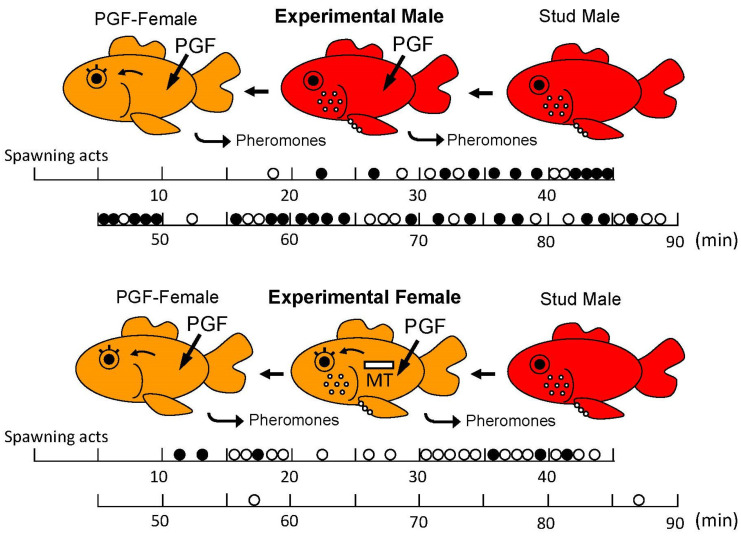
Sexual plasticity of goldfish behavior. Upper: A sexually mature experimental male injected with PGF (prostaglandin F_2α_) and placed with a PGF-injected female and a sexually mature stud male in an experimental aquarium for 90 min. The experimental male performed male-typical sexual behavior with the PGF-injected female and also performed female-typical sexual behavior with the stud male. Each open circle on the *X*-axis indicates one male-typical spawning act and each solid circle indicates female-typical spawning act. In total, the experimental male performed 31 times of male-typical acts and 19 times of female-typical spawning acts during 90 min. Lower: A sexually mature female was implanted with a capsule containing methyltestosterone (MT) a few days before the experiment and injected with PGF on the day of experiment. This experimental female was placed with a PGF-injected female and a sexually mature stud male. The experimental female performed female-typical sexual behavior with the stud male and also performed male-typical sexual behavior with the PGF-injected female. The experimental female performed 6 times of male-typical acts and 20 times of female-typical spawning acts during 90 min [47].

Later, at the time of ovulation (follicular rupture), circulating levels of PGF, a lipid involved with ovulation, jump close to hundred-fold in female goldfish, which also start clearing it via their urine [75]. PGF is released along with several metabolites, including 15-keto-prostaglandin F_2α_ (15K-PGF), which seems to be the primary component and whose release rate can approach 1 μg/h via the urine which is pulsed [75,136] (see below). 15K-PGF is about 100 times more potent than PGF itself and has its own olfactory receptors [75,108]. Interestingly, PGF is produced by ovulated eggs located in the oviduct and serves to stimulate female receptivity and behavior, thereby causing ovulated females to court males (which they discern by AD release which increases with male sexual activity (Figure 8 and Figure 9)) [3] and release eggs while they are fertile [75]. PGF-injection can stimulate totally normal spawning behavior and pheromone release in both non-ovulated females and males (Figure 10). Male (but not female) goldfish olfactory systems are extremely sensitive to PGF, as well as one of its primary metabolites, 15K-PGF [110]. Together, these products and the species-identifying cue serve to attract males and stimulate their attention [3]. Males are discerned by females by the large amounts of pheromonal AD they release (50 ng/h) which also rises greatly with sexual activity, and can reach 1 μg/h, very likely in response to the surge in LH observed in sexually active males [77]. A role for LH is indicated by the fact that HCG injection can also stimulate an increase in plasma AD levels (Iwata, unpublished) and release of AD by males [77]. The source of AD is likely the testis and possibly small amount by the interrenal gland. A physiological role of AD in males remains to be elucidated. LH release in spawning males is first triggered by DHP and DHP-S and further stimulated by PGF pheromones and spawning behavior itself [4]. PGF and 15K-PGF-sensitive olfactory neurons in male goldfish run to the olfactory bulb where they interact with unique components of the neuroendocrine system but not the terminal nerve [4]. Urinary PGF release is modulated and pulsed by females according to social context [136] and responses to it are mediated by novel olfactory receptors on novel neurons [142]. Olfactory sensitivity of the olfactory epithelium to PGF is sexually dimorphic although sensitivity to sex steroidal odorants is not [143]. As mentioned previously, this pheromone is so important to males that if rendered anosmic, they usually fail to spawn [8,10].

In females, olfaction is also important for the occurrence of sexual behavior. Female-typical sex behavior induced by PGF injection was completely blocked by nasal occlusion (blocking access to the olfactory nerve). It is not known which olfactory cues are essential for the occurrence of female-typical sex behavior although water-borne AD is attractive to sexually receptive females so likely it is a component [3]. Interestingly, olfactory tract section did not inhibit the occurrence of PGF-induced female-typical sex behavior [8,10]. When females were treated with nasal occlusion and olfactory tract section simultaneously, the PGF-injected fish actively performed sexual behavior. Although these results of experiments using nasal occlusion and olfactory tract section appear contradictory, one possible interpretation is that blocking olfactory cues in females exerts strong inhibition on sexual behavior mediated by the olfactory pathway from the olfactory epithelium to the telencephalon via the olfactory bulb [8,10]. It is possible that olfactory tract section blocked the pathway and removed the inhibitory signaling for the behavior. The olfactory bulb is well known to have inhibitory components [144]. Although olfactory tract-sectioned females do not receive olfactory information, the inhibition of olfactory pathway was removed, and the female performed sexual behavior in response to injected PGF. It is also possible, that without olfactory cues including AD, anosmic females cannot easily discern males so spawning behavior is confused and erratic.

#### 6.2.3. The Goldfish as a Model Pheromone System for Other Fishes

The goldfish hormonal pheromone system is the best understood amongst the fishes. It is notable that the key components of goldfish hormonal sex pheromone, AD, DHP and PGF, are common hormones in many teleost fishes. Not surprisingly, a wide variety of studies have now shown that approximately a dozen families of fish use these same components or their related products as sex pheromones themselves [4]. This work has been extended to the field [130,145]. Species specificity appears to lie in nuanced mixture composition as well as the species-identifying cue along with physical context—but much remains to be discerned [3,4].

Recent studies of communication in the Ostariophysans show how the addition of few steroids to olfactory repertoires/pheromonal mixtures over evolutionary time can drive the evolution of species-specific pheromone systems in fish [131]. Even greater variation in the olfactory sensitivity of fish to PGF products than to steroidal products is observed between taxonomic groups as might be expected because species specificity at spawning is especially important [4,146]. Interestingly, new studies of South American cichlids are also showing that while PGF may be highly conserved as a hormone that drives sexual behavior in ovulated female fish including the goldfish, its use as a pheromone is not [147].

Amongst species that use the some of the same hormonal components as the goldfish are other the Cyprinidae including the zebrafish, an important biomedical model, and several invasive carps [131,139] for which pheromones are now being explored as control agents. Given its relatively large size and ease of care, the goldfish is a very useful and relevant model to understand how neural and neuroendocrine systems function in vertebrates and fish in particular, as well as how chemical signaling systems have evolved.

### 6.3. Involvement of Estrogen and Androgen in Goldfish Sexual Behavior

Unlike mammals in which estrogens and progestins have key roles in female reproductive behavior [4], ovarian sex steroids are not essential for female-typical sex behavior in female goldfish. In contrast, PGF triggers female-typical sex behavior in ovulated females and other immature fish including ovariectomized female goldfish if injected [148]. Administration of sex steroids does not enhance the PGF-induced female-typical sex behavior. Androgen capsule-implanted females show male-typical sex behavior in response to the PGF pheromone [76].

When an E2 capsule is implanted into sexually mature males, plasma T and KT levels decrease. These E2-implanted males lose their tubercles, and then show a decrease in expressible milt, and finally a decrease in male-typical sex behavior in 14 to 15 weeks. The inhibitory effects of E2 on male-typical behavior is thought to be caused by a decrease in androgen production in the testis rather than direct action of E2 on the brain [35]. Studies using different androgens show that while sensitivity of olfactory PGF is almost entirely attributable to circulating androgen in male goldish along with certain aspects of male arousal, not all aspects of male-typical can be fully explained [143]. Endocrine disrupting chemicals which have estrogenic activity may suppress male-typical sex behavior in wild fishes including goldfish [38,149].

## 7. Sexual Bipotentiality in the Goldfish

### 7.1. Sexual Bipotentiality of Behavior and the Olfactory Sense

#### 7.1.1. Behavior

The goldfish is a non-sex changing gonochoristic species. Nevertheless, treating goldfish with hormones can induce sexual behavior of either the original gender (i.e., homo-typical) or opposite gender (hetero-typical gender) (Figure 9). Administrating PGF can immediately induce totally normal female-typical sex behavior in female as well as male goldfish within several minutes of injection [8]. Remarkably, sexually mature males injected with PGF will also perform normal female-typical sex behavior including the egg releasing act with other males (although no actual egg release occurs.) The latency of behavior and frequency of egg releasing act per minute is almost the same for PGF treated males and females [8]. Additionally, PGF-injected males that performed female-typical sex behavior do not lose their ability to perform male-typical sex behavior [47]. In particular, it was also shown that sexually mature male goldfish can perform male-typical and female-typical sex behavior interchangeably in the span of minutes: they are sexually bipotential. (Figure 10). These results indicate that the concept of brain sex differentiation in mammals cannot be directly applied to the goldfish [150]. In mammals, brain sex differentiation occurs during neonatal period: presence of T produced by the fetal testis usually masculinizes the fetal male brain and the fetal ovary does not produce T or E2 which will result in a feminine brain. There is no evidence that one’s postnatal social environment plays a crucial role in the development of gender identity or sexual orientation in humans [150]. Mammalian brains generally have a single neural circuit which regulates either male- or female-typical sexual behavior but in goldfish, males have neural circuits which regulate both male and female-typical sexual behaviors.

In contrast to the complete sexual bipotentiality of male goldfish, female goldfish have less plasticity with respect to sexual behavior. Androgen treatment of females induces male-typical sex behavior in response to PGF-injected females, but the behavioral activity is less robust than in natural mature males [47,143]. Interestingly, administration of androgens to females increases the peripheral olfactory sensitivity to PGF pheromone of the females to levels that can be greater than males. However, unlike complete sexual bipotentiality of the male brain, sexual bipotentiality of the female brain is incomplete. Although androgen treated females became sensitive to PGF pheromone, the neuronal pathways from the olfactory epithelium to and in the telencephalon seem to be different between males and females. However, as in males, androgen-treated females which performed male-typical sex behavior did not lose the ability to perform female-typical sex behavior.

**Figure 11:**
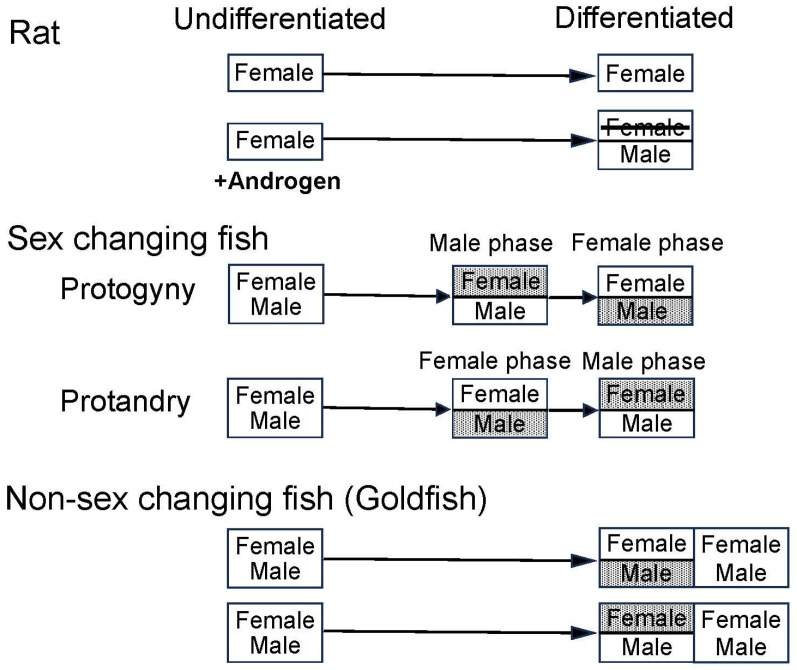
A hypothesis of sexual bipotentiality of the brain in teleost fishes (adapted from Munakata and Kobayashi, 2010 [2]). In the rat, the undifferentiated brain sex is believed to normally be female but in the presence of androgen or estrogen during the perinatal period, the brain develops neural systems which regulate male functions and inhibit female functions (crossed out area). Teleost fish, including goldfish, on the other hand, appear to possess a sexually bipotential brain. When a protogynous hermaphroditic fish is in the female phase, the female portion of the brain is activated and the male portion is quiescent (shaded area). At the time the individual starts to behave as a male, the male portion of the brain is activated, and the female portion becomes quiescent (shaded area). Physiological or external factors (age, social status, etc.) that regulate the sex change vary among species. Although gonochoristic teleosts normally use only the brain areas controlling homo-typical behaviors during their lifetime, the brain areas controlling heterotypical behaviors can be activated by hormonal treatments in some species. See text for details.

Such sexual bipotentiality has been observed in other fish species. Male-typical sex behavior can be induced in females by androgen in the medaka [151] and mosquitofish, *Gambusia affinis affinis* [152] and by cortisol in mosquitofish [153]. Since the goldfish is a gonochorist, it does not normally perform hetero-typical sexual behavior. However, it is interesting that goldfish have neural circuits which can regulate male-typical and female-typical sexual behaviors. Under natural conditions, the neural circuits for hetero-typical behavior are quiescent both in males and females, but by hormone administration, these neural circuits can be activated (Figure 11). We do not know why the goldfish has a sexually bipotential brain and what its biological significance might be. However, it does make the goldfish, and its relatives, susceptible to endocrine disrupters [152]. For this reason, study of sexual bipotentiality in fish is important for conservation.

#### 7.1.2. Olfaction

The olfactory sense, or cranial nerve #1, is critical for sexual behavior and LH surge in males (in addition to feeding; [8,41,42,144]). As mentioned above, female goldfish release DHP and a few other steroids as a preovulatory pheromone. Sexually mature male goldfish with high plasma androgen levels have a very sensitive peripheral olfactory system to DHP and other sex steroids and show LH surge stimulated by DHP [94]. However, while the female olfactory epithelium senses DHP nearly as well as males, they do not usually show an LH surge even exposed to DHP although there are indications that this may also occur in ovulatory females [9,137]. Interestingly, females treated with androgens do show a LH surge in response to DHP like males [154]; androgens seem to act on multiple olfactory centers of the brain and periphery.

Mature males also have a very sensitive and sexually dimorphic peripheral olfactory system which detects both PGF and 15K-PGF, causing them to perform male sexual behavior. However, females do not sense PGF or 15K-PGF well or perform male sexual behavior [143]. Nevertheless, when females are treated with androgens, the female peripheral olfactory system becomes highly sensitive to water-borne PGFs, and the fish will start to perform male sexual behavior although it is incomplete and weak [143]. Androgens thus seem to act on both the peripheral olfactory system and the brain, and in different manners. Nevertheless, olfaction is important for female goldfish to perform female-typical sex behavior. Nasal occlusion completely blocked the occurrence of the female-typical sex behavior in PGF-injected females. However, olfactory tract section did not block the occurrence of female-typical sex behavior in PGF-injected females as described before; this is a female-typical olfactory system [8]. Female-typical sex behavior can also be induced in male goldfish by PGF injection, and nasal occlusion completely blocked the female-typical sex behavior in the males as did females [10,46]. After olfactory tract section or combination of nasal occlusion and olfactory tract section did not inhibit the occurrence of female-typical sex behavior in males. These results indicate that males may have the same olfactory pathway for female-typical sex behavior as do females and support the idea of brain sexual bipotentiality of male goldfish. The biological and evolutionary significance of bipotentiality remains unknown.

### 7.2. Sexual Bipotentiality of LH Surge

The ovulatory LH surge occurs in females when plasma T levels are high and external factors such as water temperature rise, triggering an LH surge which is synchronized with photoperiod. So far, we have not succeeded in inducing an ovulatory surge in males with steroid treatment. However, the male-typical ovulated pheromone (DHP)-induced LH surge could be induced in female goldfish after implantation of androgen capsules. Normally, females do not show a male-typical LH surge and do not respond to DHP pheromone when kept with females ovulated. Interestingly, androgen-implanted females showed a male-typical LH surge when held with ovulatory females but did not ovulate [154]. Interestingly, androgen implantation did not inhibit the occurrence of the ovulatory LH surge in females because some androgen-implanted females ovulated during the experimental period.

### 7.3. Sexual Bipotentiality of Secondary Sexual Characteristics and Gonadal Differentiation

Sexually mature males develop male secondary characteristics including tubercles on their opercula and the anterior edges of their pectoral fins. Tubercle growth can also be stimulated in sexually immature goldfish and female goldfish by administering androgens [76]. When KT capsules are implanted into sexually mature females after partial ovariectomy, testicular tissue developed in the remaining ovarian tissue [118]. Even after gonadal maturation, ovaries remain bipotential as in hermaphroditic fishes.

## 8. The Future of Goldfish as a Model and Conclusions

As elaborated above, the goldfish is an extremely well understood, interesting, and valuable model for understanding all aspects of fish biology, and reproductive physiology in particular. This species is easily kept and sampled and closely related to other important fishes including the zebrafish, the leading vertebrate model for genetics and development, and the carps which are important for food. The size and resilience of goldfish to surgical procedures have rendered it very useful to neurobiological study. Indeed, studies of the goldfish have been instrumental in developing a detailed understanding of hormones and cellular processes in all fish as well as several neuroendocrine procedures to stimulate maturation and spawning in captive carps [155]. We fully expect this work to continue.

The goldfish has also been an invaluable model for understanding pheromonal function and chemical ecology in fishes. Insights from the goldfish hormonal pheromone system have proven to be immediately transferable to the common carp *Cyprinus carpio* [156], and the silver carp *Hypophthalmichthys molitrix* [131], which use slightly different mixtures of hormonal products as both priming and releasing sex pheromones along with a species-identifying cue [3]. It is now possible to evaluate how pheromone systems may have evolved in fishes and become specialized by comparing goldfish with related carps [131]. Goldfish studies have also inspired similar work on chemical communication in many taxonomic groups, cichlids and salmonids in particular [4,12]. It is also now clear that hormonal pheromones are used by many species and that their identities and functions have been molded by a combination of ecological pressure and genetic constraints and adaptation.

Work on goldfish reproduction has also been of practical significance because two close relatives, common carp and silver carp, are highly invasive species in many parts of the world including North America. The common carp in particular has a nearly identical reproductive biology which includes spawning in floating plants at daybreak [157,158]. Fishery managers have been able to take lessons from goldfish laboratory work, and the role of pheromones in particular, to develop control strategies for this invasive species [32,130,131].

In addition to continuing to serve as an important biomedical model for understanding circulating hormone functions and neurobiology (olfaction, sexual bipotency), the goldfish has recently become of interest in its own right: it is now being reported as an invasive species in several countries [159,160,161,162,163]. When released into certain environments (often those with low oxygen, high turbidity, and few predators), goldfish can reproduce and thrive. Here they, unfortunately, affect the aquatic ecosystems and decrease the number of native species by changing the ecosystem. We hypothesize that these feral, invasive populations might be controllable using sex pheromones (or PGF-injected fish; see [130]) and artificial plants [164] as attractants. Mating disruption might also be possible using PGF-injected immature fish. Many environmentally safe options appear possible.

In conclusion, the goldfish is a uniquely valuable biomedical, ecological, and evolutionary model which has enormous promise to continue to help advance our understanding of animal reproduction, physiology, management and conservation in many ways. We hope that this review has enhanced this promise.

## Figures and Tables

**Table 1:** Molecular type of GnRHs.

Molecular Type	Original Name	Amino Acid Sequence
GnRH1	Mammalian type GnRH	pGlu-His-Trp-Ser-Tyr-Gly-Leu-Arg-Pro-Gly-NH2
GnRH1	Chicken I-type GnRH	pGlu-His-Trp-Ser-Tyr-Gly-Leu-Gln-Pro-Gly-NH2
GnRH1	Catfish type GnRH	pGlu-His-Trp-Ser-His-Gly-Leu-Asn-Pro-Gly-NH2
GnRH1	Seabream type GnRH	pGlu-His-Trp-Ser-Tyr-Gly-Leu-Ser-Pro-Gly-NH2
GnRH1	Herring type GnRH	pGlu-His-Trp-Ser-His-Gly-Leu-Ser-Pro-Gly-NH2
GnRH1	Medaka type GnRH	pGlu-His-Trp-Ser-Phe-Gly-Leu-Ser-Pro-Gly-NH2
GnRH2	Chicken-II type GnRH	pGlu-His-Trp-Ser-His-Gly-Trp-Tyr-Pro-Gly-NH2
GnRH3	Salmon type GnRH	pGlu-His-Trp-Ser-Tyr-Gly-Trp-Leu-Pro-Gly-NH2

Adapted from Okubo and Nagahama. 2008 [84].

**Table 2:** Distribution of GnRH in fish brain area.

Species	Preoptic Area	Midbrain Tegmentum	Olfactory Bulbs
Medaka	GnRH1 (medaka type)	GnRH2 *	GnRH3 (salmon type)
Red seabream	GnRH1 (seabream type)	GnRH2	GnRH3 (salmon type)
Masu salmon	GnRH3 (salmon type)	GnRH2	GnRH3 (salmon type)
Japanese eel	GnRH1 (mammalian type)	GnRH2	GnRH1 (mammalian type)
African catfish	GnRH1 (catfish type)	GnRH2	GnRH1 (catfish type)
Goldfish	GnRH2, GnRH3 (chicken-II type, salmon type)	GnRH2	GnRH2, GnRH3 (chicken-II type, salmon type)

* GnRH2, chicken-II type. Adapted from Okubo and Nagahama. 2008 [84].

## Data Availability

No new data were created or analyzed in this study. Data sharing is not applicable to this article.

## Abbreviations

AD	androstenedione
CCK	cholecystokinin
DHP	17,20β-dihydroxy-4-pregnen-3-one
DHP-S	17,20β-dihydroxy-4-pregnen-3-one sulfate
E2	estradiol-17β
FSH	follicle-stimulating hormone
FSH-RH	follicle-stimulating hormone-releasing hormone
GnIH	gonadotropin inhibitory hormone
GnRH	gonadotropin-releasing hormone
GTH	gonadotropin
HCG	human chorionic gonadotropin
HPG	hypothalamus–pituitary–gonad (axis)
KT	11-ketotestosoterone
LH-RH	luteinizing-hormone releasing hormone
LH	luteinizing hormone
MIS	maturation-inducing steroid
MPF	maturation-promoting factor
PGF	prostaglandin F_2α_
15K-PGF	15-keto-prostaglandin F_2α_
rFSH	recombinant follicle-stimulating hormone
rGTH	recombinant gonadotropin
rLH	recombinant luteinizing hormone
T	testosterone
TSH	thyroid-stimulating hormone
